# Supplementary material for: The Broad-Spectrum Endolysin LySP2 Improves Chick Survival after Salmonella Pullorum Infection
Source: Viruses. 2023 Mar 24;15(4):836. doi: 10.3390/v15040836 (PMC10142873; doi:10.3390/v15040836)
Supplement: Supplementary file 1 [file viruses-15-00836-s001.zip › viruses-2226996-supplementary.pdf]

Optimal eukaryotic expression conditions of LySP2

Recombinant yeast expression strain X33-pPICZ-LySP2 was used to express LySP2, and 1.5% (v/v) methanol (Figure S1A) was added every 12 hours to obtain the highest expressed endolysin at 29 ° C (Figure S1B). Get maximum endolysin concentration after 96 hours (Figure S1C).

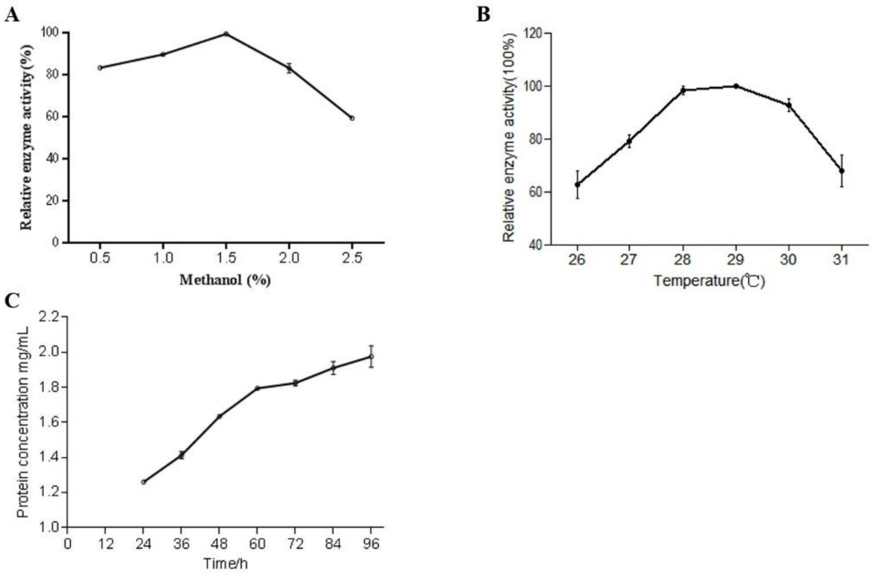

Figure S1. Optimal Induction Conditions of LySP2. (A) Optimal methanol concentration. (B) Optimal induction temperature. (C) Induction time.

Description	Max Score	Total Score	Query Cover	E value	Per. Ident
lysin [Salmonella phage YSP2]	337	337	100%	6e-117	100.00%
lysozyme [Salmonella enterica]	335	335	100%	4e-116	99.39%
lysin [Salmonella phage vB_SenS_PHB07]	334	334	100%	7e-116	98.78%
LysN [Escherichia virus TLS]	325	325	100%	2e-112	95.73%
Phage lysin (EC # Phage lysozyme or muramidase (EC [Escherichia phage Stevie_ev116]	322	322	100%	6e-111	93.90%
lysozyme [Salmonella enterica]	319	319	98%	5e-110	95.06%
lysozyme [Salmonella enterica subsp. enterica serovar 4,[5],12:i:-]	319	319	100%	6e-110	93.90%
LysN [Salmonella virus SP126]	318	318	98%	2e-109	94.44%
SAR endolysin [Citrobacter virus Stevie]	318	318	100%	3e-109	92.68%

Figure S2. The result of blast p alignment of LySP2 amino acid

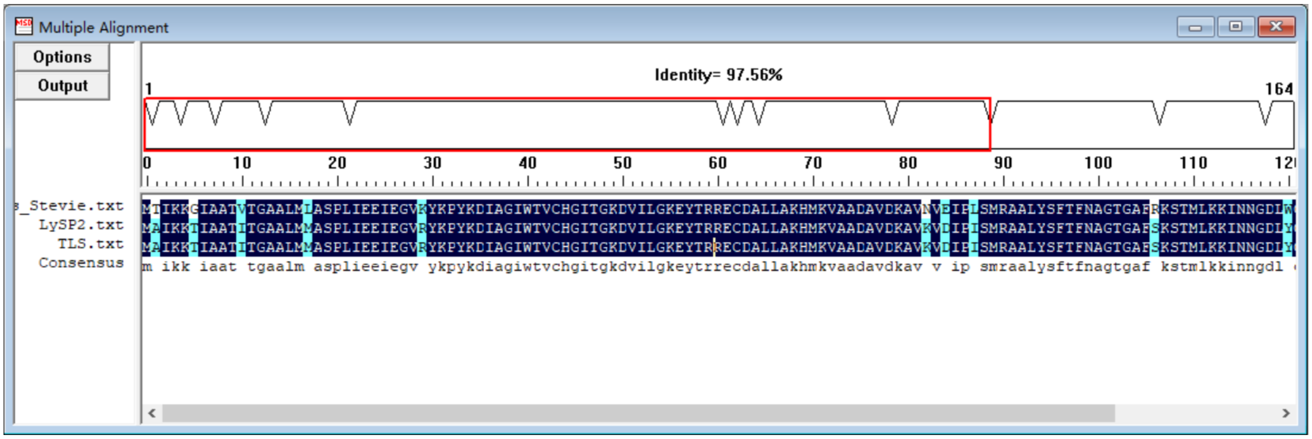

Figure S3. The result of Multiple alignments of amino acids of LySP2 and endolysin of *E. coli* phage TLS and Citrobacter phage Stevie by DNAMAN.

Express LySP2 using a prokaryotic expression system

The E. coli expression system was used to produce LySP2. A 495 bp DNA fragment containing the *lysp2* gene was amplified by PCR using phage YSP2 genomic DNA as a template, and primers LySP2-F (CGCGGATCCATGGCTATTAAAAAGACAAT) and LySP2-R (CCGCTCGAGTCATTTATTCAGATCCATTAC). Restriction endonuclease sites for *Bam*HI and *Xho*I were included at the 5′ ends of LySP2-F and LySP2-R, respectively. The purified PCR products were digested with *Bam*HI (Takara, Dalian, China) and *Xho*I (Takara, Dalian, China) and ligated into the corresponding sites of vector pET28a using T4 DNA ligase (Takara, Dalian, China) to construct recombinant plasmid pET28a -LySP2. The recombinant plasmid pET28a -LySP2 was transformed into *E. coli* BL21(DE3) pLysS for screening the positive clones called BL21- pET28a -LySP2. The empty plasmid pET28a transformed into *E. coli* BL21(DE3) pLysS called BL21- pET28a as the blank control. A single colony of the positive clones was cultured in 5 mL of LB broth containing 50ugml^{-1} kanamycin at 37 °C under constant shaking at 180 rpm until the OD600 reached 0.5.

Construction of recombinant prokaryotic expression strain BL21- pET28a -LySP2

The target gene *lysp2* was amplified by PCR (Figure. S4A) and used to construct the recombinant plasmid pET28a -LySP2. After transforming into *E. coli* BL21(DE3) pLysS to obtain the recombinant expression strain BL21- pET28a -LySP2 (Figure. S4B). Unexpectedly, when the positive recombinant strain BL21- pET28a -LySP2 was cultured to the OD600 reached 0.5, the bacterial suspension rapidly became clear (Figure S4C), and broken bacterial bodies in the culture medium produced many residues. The control strain BL21- pET28a was normal.

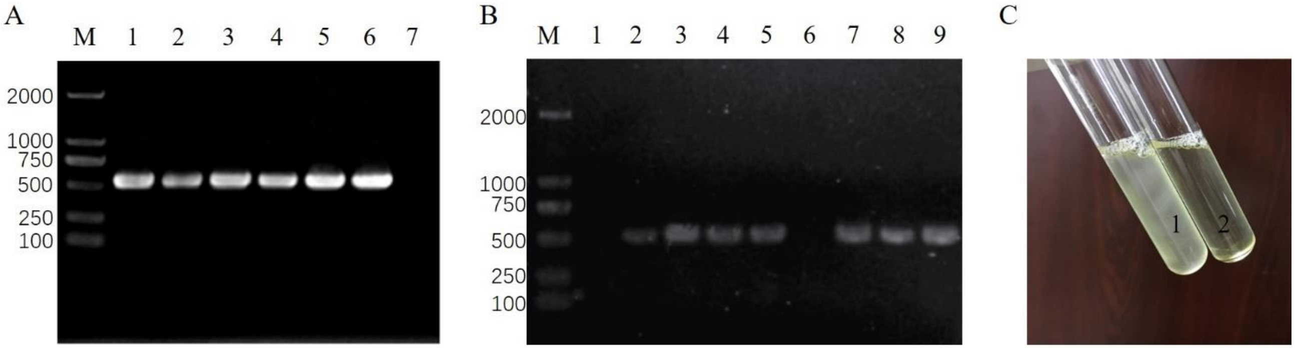

Figure S4. Construction of recombinant prokaryotic expression strain BL21- pET28a -LySP2. (A) *lysp2* PCR amplification product. Lanes: M, DL 2,000 DNA marker; 1-6, *lysp2* PCR amplification product; 7, H_2O. (B) Identification of BL21- pET28a -LySP2 by PCR. Lanes: M, DL 2,000 DNA marker; 1, H_2O; 2-5, *lysp2* PCR amplification product; 6, BL21- pET28a PCR amplification product; 7-9, BL21- pET28a -LySP2 PCR amplification product. (C) 1, BL21- pET28a Bacterial Culture Suspension; 2, BL21- pET28a -LySP2 Bacterial Culture Suspension.

Bacteriostatic spectrum and diameter of LySP2

The agar diffusion method showed lysing activity of LySP2 on 11 strains of test bacteria. The diameter (Table S1) of the lysed spots for each strain shows the relative lytic activity of LySP2 on them.

Table S1. Bacteriostatic spectrum and diameter of lySP2

Test strains		Diameter(mm)	
Salmonella pullorum	28.75	30.55	31.15
Salmonella pullorum Y	18.286	16.45	17.62
Salmonella typhimurium SL1344	14.8	15.86	13.25
Salmonella typhimurium SL7207	21.087	18.52	
Salmonella enteritidis CVCC514	18.125	21.153	20.73
Salmonella enteritidis SE	15.42	16.9	21.3
Escherichia coli DH5a	24.33	20.2	27.86
Escherichia coli ATCC25922	16.13	14.76	15.73
Escherichia coli K88	20.19	17.74	18.67
Staphylococcus aureus ATCC29213	6.5	6	
ATCC27853	6	6	

Safety test of LySP2

The X33-pPICZ-LySP2 fermentation product (100 μL) was uniformly applied to LB solid medium and cultured at 37 °C for 18-24 h to detect bacterial growth. Twenty specific pathogen-free (SPF) chicks (Beijing Melia Verton Laboratory Animal Technology Co., Ltd., Beijing, China) were randomly divided into groups A and B, with 10 chicks in each group. Group A chicks were given 500 μL of purified X33-pPICZ-LySP2 fermentation product (60 μg/mL). Group B chicks were given 500 μL sterile PBS as control. Observe whether the chicks are sick.

Result of the safety test

In vitro experiment: the fermentation liquid did not grow colonies on the LB agar plate after long-term cultivation, and the plate was transparent, indicating that the fermentation supernatant did not contain pathogenic bacteria.

In vivo experiment: the survival rate of the chicks given orally administered fermentation products was 100%, and the diet and activities were basically normal without any abnormalities.
